# Preparing medical students for a pandemic: a systematic review of student disaster training programmes

**DOI:** 10.1136/postgradmedj-2020-137906

**Published:** 2020-06-09

**Authors:** James Ashcroft, Matthew H V Byrne, Peter A Brennan, Richard Justin Davies

**Affiliations:** Cambridge Colorectal Unit, Addenbrooke's Hospital, Cambridge, UK; Department of Urology, Addenbrooke's Hospital, Cambridge, UK; Maxillofacial Surgery, Queen Alexandra Hospital, Portsmouth, UK; Cambridge Colorectal Unit, Addenbrooke's Hospital, Cambridge, UK; School of Clinical Medicine, University of Cambridge, Cambridge, Cambridgeshire, UK

**Keywords:** education & training (see medical education & training), medical education & training, trauma management, accident & emergency medicine

## Abstract

**Objective:**

To identify pandemic and disaster medicine-themed training programmes aimed at medical students and to assess whether these interventions had an effect on objective measures of disaster preparedness and clinical outcomes. To suggest a training approach that can be used to train medical students for the current COVID-19 pandemic.

**Results:**

23 studies met inclusion criteria assessing knowledge (n=18, 78.3%), attitude (n=14, 60.9%) or skill (n=10, 43.5%) following medical student disaster training. No studies assessed clinical improvement. The length of studies ranged from 1 day to 28 days, and the median length of training was 2 days (IQR=1–14). Overall, medical student disaster training programmes improved student disaster and pandemic preparedness and resulted in improved attitude, knowledge and skills. 18 studies used pretest and post-test measures which demonstrated an improvement in all outcomes from all studies.

**Conclusions:**

Implementing disaster training programmes for medical students improves preparedness, knowledge and skills that are important for medical students during times of pandemic. If medical students are recruited to assist in the COVID-19 pandemic, there needs to be a specific training programme for them. This review demonstrates that medical students undergoing appropriate training could play an essential role in pandemic management and suggests a course and assessment structure for medical student COVID-19 training.

**Registration:**

The search strategy was not registered on PROSPERO—the international prospective register of systematic reviews—to prevent unnecessary delay.

## INTRODUCTION

Global disasters, such as a pandemics or warfare, are events that cause a major disruption to health and social care, industry and economy, and community and education.^1^ Disasters on this scale result in substantial loss of life, and an immeasurable burden is placed on healthcare services to deliver core medical care.^1^ Disaster healthcare provision requires a collaborative approach that uses the expertise and skills of as many people as possible.

Much of what is formally taught in medical school is around the knowledge, skills and behaviours required of a physician for patients at the bedside.^2^ However, the broad training medical students receive could be applied to disaster scenarios especially if supported with adjunct specialist training. The current medical student curriculum already covers a wide range of specialties, and some may argue it is stretched. However, the rising incidence of worldwide disasters and the impact of the current coronavirus (COVID-19) pandemic has justified the need for disaster preparation training in medical students.^1^ In some respects, students with disaster training may be better suited to assist in both clinical and non-clinical roles in disaster scenarios than redeployment of senior physicians with super-specialist skills and knowledge. Curricula using multidisciplinary methods of simulation and human factors training have been proposed for implementation by the USA (Association of American Medical Colleges^3^) and Europe (Government of the Federal Republic of Germany^4^ and Research Center in Emergency and Disaster Medicine and Computer Science Applied to Medical Practice, Italy^5^). However, at present, it is recognised that there is a brief or non-existent exposure to disaster training within current medical training curricula across the world, which may leave students unprepared for an intimidating and unfamiliar setting if assisting in the healthcare workforce.^1^

The current COVID-19 pandemic is rapidly driving the need for healthcare workers in the UK.^6^ On 24 March 2020, the UK Health Secretary, Matt Hancock announced plans to introduce medical students as volunteers to the NHS in order to assist in this pandemic.^7^ In response, the British Medical Association and Medical Schools Council issued clear advice regarding medical students joining the UK healthcare workforce including ensuring correct induction, training and supervision.^8^ The aim of this study was to systematically review disaster training courses for medical students. We describe the educational structure and methodology employed, and evaluate both preparedness for disaster medicine and learning outcomes to inform the development of COVID-19-specific training programmes.

## METHODS

We adhered to PRISMA (Preferred Reporting Items for Systematic Reviews and Meta-Analyses) guidelines and recommendations for systematic reviews of observational studies.^9^

### Data sources

We searched Embase, Medline and Cochrane Central from 1996 for all articles published until 19 March 2020 evaluating training that medical students receive to prepare them for pandemics and disasters, with no language restriction. We identified 1289 articles, which we then screened for inclusion.

### Search strategy

The search was conducted using the following Medical Search Headings: ‘Coronavirus’, ‘Covid-19’, ‘SARS virus’, ‘disasters’, ‘natural disaster’, ‘major catastrophe’, ‘mass casualties’, ‘crisis event’, ‘extreme weather’, ‘disease outbreaks’, ‘infectious disease transmission’, ‘epidemics’, ‘pandemics’, ‘mass drug administration’, ‘warfare’, ‘biohazard release’, ‘chemical hazard release’, ‘radioactive hazard release’, ‘radiation exposure’, ‘radiation injuries’, ‘hazardous’, ‘waste’, ‘chemical water pollution’, ‘radioactive water pollution’, ‘medical students’, ‘medical schools’, ‘education’ with terms exploded as appropriate. The search strategy was not registered on PROSPERO—the international prospective register of systematic reviews—to prevent unnecessary delay.

### Study selection

**Figure 1:**
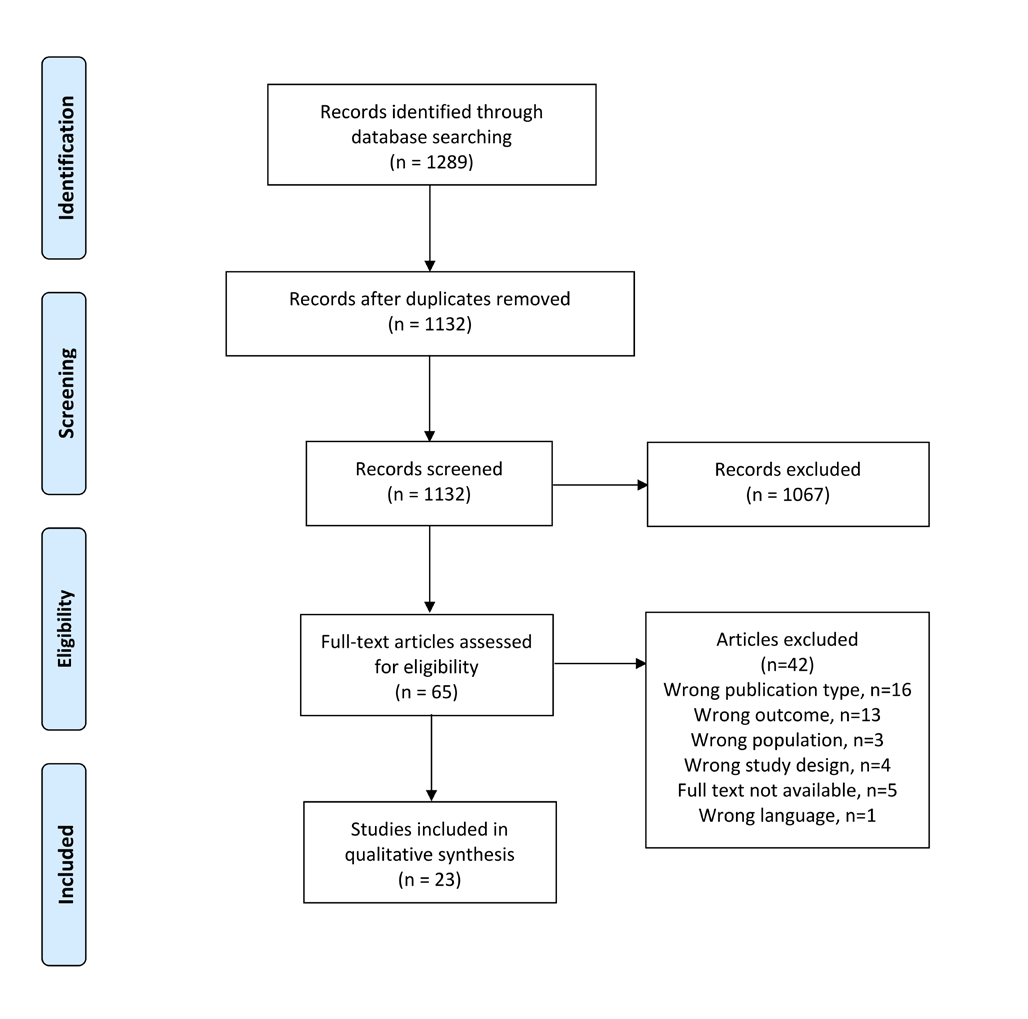
PRISMA (Preferred Reporting Items for Systematic Reviews and Meta-Analyses) search strategy flow diagram.

We selected randomised controlled trials, case–control studies and cohort studies that measured medical student training outcomes in the context of pandemics and disasters. Studies were selected only if they contained a detailed report of the training implementation and used objective precourse and/or postcourse assessments related to medical student knowledge, attitude, skills or clinical care outcomes. Importantly, if medical student outcomes were grouped with other healthcare students or professionals and not reported separately, the study was excluded. We excluded non-English language articles in order to ensure data quality, logistical training process evaluations, literature reviews, case reports, clinical trial proposals, conference abstracts, editorials, letters and articles evaluating non-medical student populations only. Pandemic infections that may be secondary issues to a disaster, but were not the primary cause, were also excluded, for example, HIV, dengue, malaria; situations where medical students were unlikely to be required to volunteer en masse, for example, active shooter situations; and interventions that were not in a disaster setting, for example, basic life support and routine clinical infection control procedures were excluded. Duplicates were removed and two reviewers (JA and MHVB) independently screened titles and abstracts using Rayyan, an online software to aid blinded abstract screening.^10^ Any discrepancies were resolved by consensus. Of the 1289 citations screened, we identified 65 articles for full text and reference review, of which 23 final studies met the inclusion criteria for data synthesis (figure 1).

### Data extraction

Two reviewers (JA and MHVB) independently extracted relevant information from each training report using standardised data extraction proforma in keeping with Best Evidence Medical Education recommendations and one author with medical training expertise (RJD) reviewed all extracted data.^11^ We recorded administrative information including authorship, institution and year of publication; training-related data including details and duration of intervention, participants and teaching methods; and quantitative and qualitative outcome measures.^11^ The quality of training intervention and risk of bias in reporting of results was assessed using the ROBINS-1 for non-randomised controlled trials.^12^

### Analysis

Meta-analysis was not performed on the training outcomes assessed due to the wide heterogeneity in training interventions and reporting of results. Descriptive analysis was performed instead. Interventions were assessed against Kirkpatrick criteria and Kirkpatrick’s levels were assigned: impact on learners’ satisfaction (level 1), changes in learners’ attitudes (level 2a), measures of learners’ knowledge and skills (level 2b), change in learners’ behaviour (level 3), changes to clinical processes/organisational practice (level 4a) and benefits to patients (level 4b).^11^

## RESULTS

### Characteristics of included studies

Twenty-three studies met the inclusion criteria, and their characteristics are displayed in table 1. The majority of studies (n=18, 78.3%) were from the USA, and other countries were Germany (n=1), Israel (n=1), Italy (n=1), Saudi Arabia (n=1) and South Korea (n=1). Five studies (21.7%) involved a multidisciplinary cohort and reported outcomes for other healthcare students and professionals, as well as outcomes for medical students individually without pooling of results.

**Table 1:** Course characteristics, structure and content

Lead author, year, Institution, country	Course structure	Medical student population	Non-medical student population	Duration of intervention	Education setting	Teaching methods
Back,^26^ 2019, Bundeswehr Hospital, Germany	Disaster medicine course which encompassed didactic and practical elements from 12 medical specialties	51, third year students	0	4 weeks	Classroom, indoor simulation, online web system	Lecture, practical skills, simulation, group discussion, computer activity, case study, self-study
Bajow,^13^ 2016, King Fahd Security College, Saudi Arabia	Emergency and disaster medicine course consisting of didactic, practical elements and field trips	29, fourth to sixth year students	0	2 weeks	Lecture hall, classroom, indoor simulation, distance learning, field trip	Lecture, simulation, role play, group discussion, video, case study, observation
Carney,^27^ 2011, Case Western Reserve, Harvard, Colorado and Vermont Colleges of Medicine, USA	Pandemic influenza courses from four universities consisting of didactic sessions followed by role play and simulated exercises	CWRU=NK, first year students HMS=729 first year students CU=146 fourth year students UVM=71 clerkship year	0	1 day	Lecture hall, classroom, indoor simulation, online web system	Lecture, simulation, role play, group discussion, computer activity
Chernock,^28^ 2019, Rutgers New Jersey Medical School, USA	Haemorrhage control tourniquet teaching with hands on practical experience on manikins	359, first to fourth year students	0	2 days	Classroom, indoor simulation	Simulation, group discussion, interactive activity, video and podcast, self-study
Goolsby,^15^ 2014, University of the Health Sciences, USA	Large-scale high-fidelity combat casualty training preceded by a combat medical skills course	91, first year military students	0	10 days	Classroom, indoor simulation	Practical skills, simulation, handout, self-study
Ingrassia,^29^ 2014 CRIMEDIM, Italy	Nationwide course on disaster medicine delivered to 21 medical schools in Italy	524, fourth to sixth year students	0	1 week	Classroom, indoor, simulation, distance learning, online web system	Lecture, interactive activity, simulation, computer activity, case study, self-study
Kaji,^30^ 2010, University of California, USA	Disaster medicine elective consisting of didactic elements, interactive exercises, and observation of disaster planning and drills	6, fourth year students	0	2 weeks	Classroom, field trip	Lecture, interactive activity, practical, observation
Lei,^31^ 2019, UTHealth McGovern Medical School, USA	Haemorrhage control tourniquet teaching followed by hands on practical experience	123, third year students	287 school nurses, 68 interdisciplinary, 77 general public	1 day	Classroom, indoor simulation	Lecture, simulation, group discussion
Lin,^32^ 2009, University of Illinois, USA	Bag-valve-mask training in disaster setting consisting of didactic sessions followed by hands on practical	31, second and fourth year students	0	1 days	Lecture hall, classroom, indoor simulation	Lecture, practical skills, simulation
Marcus,^33^ 2019, University of Toledo, USA	Haemorrhage control tourniquet teaching with low fidelity hands on practical experience	107, first year students	0	1 day	Classroom, outdoor simulation	Lecture, simulation, group discussion
Marshall,^34^ 2008, University of Hawaii School of Medicine, USA	Pandemic problem-based learning course	3, not stated	2 nursing, 1 public health, and 1 social work student and 2 social workers	2 weeks	Classroom	Interactive activity, group discussion, case study
Myong,^35^ 2016, The Catholic University of Korea, Republic of Korea	Respiratory protective equipment fitting education programme	50, senior students	0	Not stated	Classroom	Demonstration
Padaki,^36^ 2018, Department of Emergency Medicine, Christiana Care Health System, USA	Didactic lecture and simulated cases of in-flight medical emergencies course	18, 3 third year and 15 fourth year students	0	2 weeks	Simulation	Lectures, interactive activity, simulation
Parrish,^37^ 2005, The Texas A&M College of Medicine, USA	Military-based lecture and experiential element course	72, second year students	0	4 days	Classroom	Lectures, interactive activity
Patel,^38^ 2016, Case Western Reserve University School of Medicine, USA	Online disaster preparedness curriculum consisting of four modules	132, first to fourth year students	0	Not stated	Online web system	Computer activity
Rivkind,^39^ 2015, Hadassah-Hebrew University Medical Center, Israel	Resuscitation, procedures and decision-making course in trauma disaster management	490, not stated	0	2 weeks	Outdoor simulation	Lectures, interactive activity, simulation, group discussion case study
Scott,^40^ 2010, Medical University of South Carolina, USA	Didactic training scenario and two simulated hazardous material and mass casualty training exercises	61, fourth year students	0	1 day	Classroom, outdoor simulation	Lectures, interactive activity, simulation
Scott,^41^ 2012, Medical University of South Carolina, USA	Course revolving around three small group exercises and multi-actor clinical disaster scenarios	10, not stated	17 doctors, nurses or emergency managers	1 day	Classroom, online web system, outdoor simulation	Lectures, interactive activity, simulation
Scott,^14^ 2013, Medical University of South Carolina, USA	Three small group exercises and multi-actor clinical disaster scenarios	24, fourth year students	7 doctors, 7 nurses and 1 emergency manager	1 day	Classroom, online web system, outdoor simulation	Lectures, interactive activity, simulation
Silenas,^16^ 2008, The Texas A&M College of Medicine, USA	Small group role-playing session and debriefing course	69, second year students	20 veterinary or public health students	1 week	Classroom	Group discussion
Vincent,^42^ 2008, Telehealth Research Institute, University of Hawaii, USA	Three short podcasts followed by an immersive virtual reality-based exercise	24, first to fourth year students	0	1 day	Online web system, virtual reality simulation	Computer activity, interactive activity, podcast
Vincent,^43^ 2009, Telehealth Research Institute, University of Hawaii, USA	Four short podcasts followed by a manikin (SimMan)-based exercise	21, first to fourth year students	0	1 day	Online web system, virtual reality simulation	Computer activity, interactive activity, podcast
Wiesner,^44^ 2018, Georgetown University School of Medicine, USA	Didactic lectures and hands-on skills workshops in resuscitation	81, not stated	0	1 day	Classroom	Lectures, interactive activity, simulation

The course structures and learning objectives were grouped into three categories: broad concepts in disaster medicine (n=12, 52.2%), trauma or haemorrhage mass casualty management (n=8, 34.8%), or influenza pandemic management, airborne viral management or personal protection (n=3, 13%). The length of studies ranged from single day teaching to 4-week boot camps, and the median length of training was 2 days (IQR=1–14). The majority of training interventions used traditional didactic lectures with simulative or experiential teaching methods, with 12 courses (52.2%) containing lectures and simulation. Of the simulation experiences, four courses (17.4%) contained outdoor actor-based mass casualty simulation. Multimedia approaches were used in eight courses (34.8%) as an adjunct to training, often precourse, in order to efficiently provide material to attendees. Problem-based learning or case-based learning was used as a predominant feature in five courses (21.7%) in a classroom setting with or without other teaching methods. No courses involved only didactic teaching methods, which is reflective of the learning objectives of disaster medicine preparedness.

### Study design and quality assessment

All 23 included studies were prospective cohort studies measuring the impact of their training intervention postcourse evaluation, with 18 using precourse evaluation for comparison as displayed in table 2. The majority used subjective assessments of knowledge or preparedness in disaster medicine (n=20, 87.0%) with nine studies (39.1%) using objective measures. There was a wide range in number of medical students attending the courses, with the median number of participants being 61 (IQR 24–123). However, five studies did not clearly describe their medical student population, either omitting total number of participants or seniority of students. Only two studies^13^  ^14^ reported data that assessed longitudinal learning beyond the year of course implementation. Common limitations of study design included training being limited to a single institution, studies which were excluded due to being randomised controlled trials evaluating the efficacy of different teaching methods or simulation technology, or studies excluded due to reporting medical student outcomes pooled with outcomes of other healthcare student or professionals.

**Table 2:** Precourse and postcourse test outcomes

Lead author, year, Institution, country	Medical students assessed	Outcome	Precourse test outcome	Postcourse test outcome	Measure	P value
Back,^26^ 2019, Bundeswehr Hospital, Germany	51	Overall knowledge of disaster medicine assessed by examination	56%, 48–50	72%, 64–76	Median, IQR	<0.001
Bajow,^13^ 2016, King Fahd Security College, Saudi Arabia	29	Overall knowledge of disaster medicine assessed by examination	41.0%, 6.3	67.7%, 7	Mean, SD	<0.0001
Ingrassia,^29^ 2014 CRIMEDIM, Italy	524	Overall knowledge of disaster medicine assessed by examination	39.5%,12.9	82.9%, 17.6	Mean, SD	<0.01
		Accuracy of triage	45%	78%	Mean	<0.01
Lei,^31^ 2019, UTHealth McGovern Medical School, USA	123	Willingness to help a bleeding volunteer by Likert type five-point self-reported response	93%	99%	Mean proportion agree/strongly agree	–
		Preparedness to help a bleeding volunteer by Likert type five-point self-reported response	19%	98%	Mean proportion agree/strongly agree	–
		Overall knowledge of disaster medicine assessed by examination assessed by obtaining a pass mark of >60%	73%	100%	Pass rate (%)	–
Marcus,^33^ 2019, University of Toledo, USA	97	Overall knowledge of tourniquet application assessed by examination by Likert type five-point response	2.3, 2.0–2.5	4.4, 4.2–4.5	Mean, 95% CI	<0.001
Marshall,^34^ 2008, University of Hawaii School of Medicine, USA	3	Overall knowledge of bioterrorism preparedness by Likert type five-point self-reported response	1.4	3.6	Mean	–
Myong,^35^ 2016, The Catholic University of Korea, Republic of Korea	17	Pass rate for fit test for respiratory protection by observational measurement	30%	74%	Pass rate	<0.001
Padaki,^36^ 2018, Department of Emergency Medicine, Christiana Care Health System, USA	18	Knowledge based quiz of in-flight medical emergencies	11.3, 1.5	13.1, 2.1	Mean, SD	0.001
Parrish,^37^ 2005, The Texas A&M College of Medicine, USA	72	Precourse and postcourse assessment testing knowledge towards bioterrorism as score	8.6	10.5	Mean	<0.001
Patel,^38^ 2016, Case Western Reserve University School of Medicine, USA	50 pre, 49 post	A precourse and postcourse five-point Likert type test was undertaken to assess familiarity with acronyms	26.0%	87.6%	Mean proportion	–
	50 pre, 54 post	A precourse and postcourse five-point Likert type test was undertaken to assess self-assessed preparedness for a disaster	6.0%	58.0%	Mean proportion agree/strongly agree	–
Rivkind,^39^ 2015, Hadassah-Hebrew University Medical Center, Israel	108	Precourse and postcourse knowledge-based multiple choice questions	54.0%, 12.7%	68%, 10.2%	Mean, SD	–
Scott,^40^ 2010, Medical University of South Carolina, USA	30 (2008)	Precourse and postcourse Likert type 5 point scale assessing subjective knowledge of disaster medicine concepts	1.9	3.8	Mean	–
	31 (2009)	Precourse and postcourse Likert type 5 point scale assessing subjective knowledge of disaster medicine concepts	2.5	4.9	Mean	–
Scott,^41^ 2012, Medical University of South Carolina, USA	10	Precourse and postcourse Likert type five-point assessment testing knowledge and subjective skill towards emergency preparedness	30%	80%		–
Scott,^14^ 2013, Medical University of South Carolina, USA	17	Participants undertook a precourse and postcourse 24 mark assessment which measured trainees’ discrete knowledge	10.6, 3.2	17.8, 2.0	Mean, SD	<0.01
	17	Participants undertook a precourse and postcourse 1 to 100 analogue scale assessment which measured trainees’ subjective knowledge	24.6%, 15.2%	71.7%, 12.2%	Mean, SD	<0.01
	17	Participants undertook a precourse and postcourse 1 to 100 analogue scale assessment which measured trainees’ subjective skill	31.7%, 15.8%	75.9%, 13.5%	Mean, SD	<0.01
Silenas,^16^ 2008, The Texas A&M College of Medicine, USA	66	Precourse and postcourse Likert type three-point assessment of knowledge (1 favourable, 3 unfavourable)	1.9	1.3	Mean	–
Vincent,^42^ 2008, Telehealth Research Institute, University of Hawaii, USA	20	Precourse and postcourse Likert type five-point assessment of self-reported confidence and virtual reality feedback, in addition to between exercise measures	3.5	4.2	Mean	–
Vincent,^43^ 2009, Telehealth Research Institute, University of Hawaii, USA	21	Precourse and postcourse Likert type five-point assessment of self-reported confidence and simulation feedback, in addition to between exercise measures	3.4	4.1	Mean	–
Wiesner,^44^ 2018, Georgetown University School of Medicine, USA	46	Precourse and postcourse assessment of knowledge out of a total of 10 marks	5.3, 1.1	8.0, 1.0	Mean, SD	–

### Study evaluation and main findings

Of the studies included in this review, 18 studies in total measured precourse and postcourse outcomes. Of these 18 studies, knowledge was measured in 16 (88.9%), of which 10 undertook objective knowledge measurements, making this the most measured outcome. However, none of the subjective or objective measures of knowledge were previously described or undertaken with validated measures. A total of four studies measured attitude—either preparedness for disaster (n=2) or confidence in approaching a disaster (n=2) by precourse and postcourse assessment. Disaster medicine skills were subjectively measured in four studies and objectively measured in two, one being pass rates in personal protective equipment fitting and the second being accuracy of disaster triage. In these studies, validated measures were used to create scores or pass rates. Of the five studies inviting a multi-disciplinary participant group, none used validated crew resource management or teamwork training measures, as human factors types skills may be challenging for medical student cohorts to acquire. However, all five studies improved attitudes towards multidisciplinary healthcare teams. No studies included in this review recruited or involved patients in any of the course curriculum.

An evaluation of all 23 study outcomes and findings included in this review are displayed in table 3. Knowledge was evaluated in 18 studies (78.3%), 14 studies (60.9%) evaluated attitude and 10 studies (43.5%) assessed skills. No studies assessed clinical performance. Kirkpatrick criteria were then analysed for each study.

**Table 3:** Study outcome measures and main findings

Lead author, year, Institution, country	Outcome assessment	Main summarised findings	Kirkpatrick criteria measured	Kirkpatrick level
Back,^26^ 2019, Bundeswehr Hospital, Germany	Precourse and postcourse examination and questionnaire assessing disaster medicine knowledge and interest in disaster medicine preparedness assessed by questionnaire.	This disaster course significant increased knowledge of disaster medicine after course from 56% to 72% (p<0.001). Students strongly interested in disaster medicine increased after the course, from 63% to 80%. More than 80% agreed or strongly agreed they were satisfied with the course, and their increase in knowledge.	Knowledge, attitude	1, 2A, 2B
Bajow,^13^ 2016, King Fahd Security College, Saudi Arabia	Precourse and postcourse examination assessing disaster medicine knowledge. Questionnaire sent 1.5 years after course to assess perceived effect of course.	There was a significant increase in knowledge of disaster medicine after course from 41% to 68% (p<0.0001), independent of medical student year. Medical student year. Medical students once graduated felt less stressed and confident when handling emergencies 1.5 years after the course. More than 70% found the course interesting.	Knowledge, attitude	1, 2B, 3
Carney,^27^ 2011, Case Western Reserve, Harvard, Colorado and Vermont Colleges of Medicine, USA	Postcourse questionnaires assessing perceived understanding of public health system and response to public health threats, perceived understanding of successful pandemic/emergency preparedness response. Examination assessing knowledge of pandemic/emergency preparedness.	Overall, 69% agreed or strongly agreed they had a better understanding of the public health, 82% agreed or strongly agreed they had a better understanding of successful preparedness for a pandemic or emergency, and 70% correctly answered questions on pandemic/emergency preparedness after course. 100% agreed or strongly agreed that physicians are best prepared by having written plans and undertaking preparedness exercises after the course.	Knowledge, attitude	2A, 2B
Chernock,^28^ 2019, Rutgers New Jersey Medical School, USA	Postcourse questionnaire assessing perceived confidence using skills applying tourniquet.	92% agreed or strongly agreed they were confident using the tourniquet if required after course. 98% agreed or strongly agreed they had learnt the basics of bleeding control. 89% would recommend the course.	Attitude, skill	1 to 2B
Goolsby,^15^ 2014, University of the Health Sciences, USA	Postcourse questionnaire assessing perceived confidence at assessment and procedures in a combat casualty situation and perceived preparedness at managing combat casualties.	The majority of students feel more confident and better prepared to assess and perform procedures in a combat casualty situation after course. The majority of students preferred the high-fidelity simulation to their normal learning environment.	Attitude, skill	1 to 2A
Ingrassia,^29^ 2014 CRIMEDIM, Italy	Precourse and postcourse examination assessing disaster medicine knowledge. Triage accuracy was measured in a disaster simulation assessed by an examiner.	There was a significant improvement in knowledge of disaster medicine after course from 40% to 83% (p<0.01), and a significant improvement in triage accuracy in the disaster medicine simulation after course from 45% to 78% (p<0.01). The majority of students felt that disaster medicine should be part of their curriculum and evaluated the course highly.	Knowledge, skill	1 to 2B
Kaji,^30^ 2010, University of California, USA	Postcourse oral examination of disaster medicine knowledge.	All participants obtained a scores of >90% obtained on examination after course. All students rated the course 100% on a five-point Likert type scale.	Knowledge	1 to 2B
Lei,^31^ 2019, UTHealth McGovern Medical School, USA	Precourse and postcourse examination assessing disaster medicine knowledge and questionnaire assessing perceived willingness and preparedness to help bleeding individual.	All medical students passed the test on haemorrhage control after the intervention, compared with 73% prior to the course. Willingness of medical students to help a bleeding volunteer increased from 93% to 99% and preparedness increased from 19% to 98%.	Knowledge, attitude, skill	2A, 2B
Lin,^32^ 2009, University of Illinois, USA	Postcourse assessment of ability to perform bag valve mask and examination assessing knowledge of methods for ensuring adequate ventilation.	All students were able to satisfactorily perform bag-valve mask technique after the course. The majority of students knew proper positioning in non-trauma (93%) and trauma cases (72%), and ventilation rates (86%), and technique to ensure adequate seal (63%). However, only 29% knew how to assess adequate ventilation.	Knowledge, skill	2B
Marcus,^33^ 2019, University of Toledo, USA	Precourse and postcourse examination assessing tourniquet application knowledge, and questionnaire on perceived competency in applying tourniquet.	There was a significant improvement in knowledge of tourniquet application on test. Likert score increased from 2.3 to 4.4 after course (p<0.001). There was also a significant improvement in confidence when applying tourniquet (p<0.001).	Knowledge, skill	2A, 2B
Marshall,^34^ 2008, University of Hawaii School of Medicine, USA	An educationalist observed problem-based learning sessions and administered precourse and postcourse questionnaires on subjective knowledge in addition to online surveys on postcourse bioterrorism knowledge	Problem-based learning is effective in educating both medical students and community-based health professionals from different disciplines about issues related to pandemic preparedness in addition to allowing multidisciplinary communication and collaboration.	Knowledge, attitude	2B
Myong,^35^ 2016, The Catholic University of Korea, Republic of Korea	A qualitative fit testing tool (3M-FT10 kit) was used to assess for a pass fail of adequate respiratory protection fitting precourse and postcourse.	This course demonstrated effective teaching of respiratory protection fitting in medical students, with the proportion of individuals passing the test being 30% (n=15) before the programme and 74% (n=37) after. This may reduce the risk of infection in medical students working in the hospital with at risk patients and may allow medical students to teach this skill.	Skill	2B
Padaki,^36^ 2018, Department of Emergency Medicine, Christiana Care Health System, USA	Presimulation and postsimulation training questionnaires were administered based on knowledge an anonymous participant feedback was solicited for purposes of course improvement.	This course demonstrated a practical, low-fidelity simulation-based curriculum for education on in-flight medical emergencies. Simulation training significantly increased student performance, from a mean pretest score of 75.6% to a mean post-test score of 87.0%.	Knowledge	2B
Parrish,^37^ 2005, The Texas A&M College of Medicine, USA	Participants were asked to undertake precourse and postcourse surveys assessing their knowledge of bioterrorism and a post-test survey assessing their attitudes towards preparedness for bioterrorism.	After this course, students were more favourable in their attitudes towards their professional preparedness and the local/state government preparedness for a bioterrorist event which were rated with a mean Likert score of 4.4. There was a statistically significant improvement (8.6 to 10.5) in students’ knowledge of disaster preparedness	Knowledge, attitude	2A, 2B
Patel,^38^ 2016, Case Western Reserve University School of Medicine, USA	Participants undertook precourse and postcourse surveys assessing knowledge of acronyms Participants undertook precourse and postcourse surveys grading self-assessed preparedness for medical disasters.	An online elective in disaster preparedness resulted in students becoming more familiar with acronyms such as EMA (8% pre to 90% post), gaining a better understanding of organisations such as the American Red Cross (36% pre; 73% post), gaining triage knowledge (START triage 15% pre; 71% post). Similar proportions of students would volunteer in disaster scenarios precourse and postcourse (94% pre; 93% post).	Knowledge, attitude	2A, 2B
Rivkind,^39^ 2015, Hadassah-Hebrew University Medical Center, Israel	Students were observed thorough multidisciplinary debriefing based on video footage and action photographs. Students received feedback on communication and interaction under stress, triage decisions and clinical management.	Over a 3-year assessment period with data on 309 participants out of 490 in total, the mean knowledge pretest was 54% (12.7%) compared with 68% (10.2%). On an assessment scale of 1 to 20, students in the 2012 cohort scored the course highly for its general assessment, with high results in trauma knowledge gained mean 18.4 (1.2), assessment of self-preparedness 15.9 (3.1) and technical skills acquired mean 17.0 (2.4).	Knowledge, attitude, skill	1, 2A, 2B
Scott^40^ 2010, Medical University of South Carolina, USA	Students in the 2008 class took the pretest to survey basic knowledge and assess learning of the didactic material immediately before the 90 min case-based lecture, and the post-test immediately after the lectures. The 2009 class could take their pretest via an e-learning tool up to several days before the class, and the post-test was available online for 3 weeks after completion of the course.	Over a 2-year assessment period the first year cohort's post-test knowledge scored improved from 3.8/10 (below average to average) compared with 7.6/10 (average to above average) and the second year's post-test scores improved from 2.5/5 (average) before and 3.8/5 (above average). In the first year cohort he average overall rating for the experience was 4.9/5, and 100% of the respondents recommended the class for next year’s students allowing it to continue.	Knowledge, attitude	1 to 2B
Scott,^41^ 2012, Medical University of South Carolina, USA	Participants undertook precourse and postcourse assessment developed to meet learning objectives of the course. Self-assessment of personal capability and comfort to handle a disaster and multiple choice questions of knowledge and subjective skill were undertaken.	Most (70%) of the trainees considered their emergency preparedness knowledge and skill as average or below average before the training experience. After the curriculum, 100% of trainees considered their emergency preparedness knowledge and skill above average, and 90% would recommend the course to other healthcare workers.	Knowledge, skill	1 to 2B
Scott,^14^ 2013, Medical University of South Carolina, USA	Participants undertook an online precourse and postcourse assessment developed to meet the learning objectives and competencies of the course in addition to giving post-test feedback on the implementation of the course.	In discrete knowledge, subjective knowledge and skills all participants demonstrated significant improvements in their postcourse test results when compared with pre-test. Course evaluation was performed, and it was found that students would recommend this course (median 92.5%), whether the course was feasible (median 82.5%) and overall evaluation (94.5%).	Knowledge, skill	1 to 2B
Silenas,^16^ 2008, The Texas A&M College of Medicine, USA	Students answered Likert type scales to assess the extent to which the objectives and understanding of key concepts had been accomplished. Written and verbal comments from the students and facilitators about their experience were gathered.	Sixty-six medical students completed the knowledge test before and again 4 days after the Avian Influenza exercise. The lowest scores for knowledge were best and all tested knowledge areas except one (endemic influenza as a public health issue) decreased postcourse. The course received mixed ratings which overall were positive (33%), undecided (13%) and negative (54%).	Knowledge.	1 to 2B
Vincent,^42^, 2008, Telehealth Research Institute, University of Hawaii, USA	Students answered precourse and postcourse self-confidence questions on a five-point Likert scale with points labelled 'never' to 'always’ in addition to giving evaluation on the course implementation and use of virtual reality and between exercise virtual reality scores.	Students became more confident that their patients would consider them effective first responders (p=0.006), more confident in prioritising treatment (p=0.001), more confident in prioritising resources (p=0.001) and more confident in identifying high-risk patients (p=0.008). Students rated the simulation phase of the course highly and favourably rated on a Likert seven-point scale: pace (4.2±0.39 too slow/too fast), level of difficulty (4.5±0.83 too easy/too hard) and relevance (6.5±0.61 agree/disagree).	Attitude	1 to 2A
Vincent,^43^ 2009, Telehealth Research Institute, University of Hawaii, USA	Students answered precourse and postcourse self-confidence questions on a five-point Likert scale with points labelled 'never' to 'always’ in addition to giving evaluation on the course implementation and use of simulation and between exercise simulation scores.	Following this course students became more confident that their patients would consider them effective first responders (p<0.001), more confident in prioritising treatment (p<0.001), more confident in prioritising resources (p<0.01) and more confident in identifying high-risk patients (p<0.01). The students rated the simulation phase of the course highly and favourably rated on a Likert seven-point scale pace (4.2 too slow/too fast), level of difficulty (4.0 too easy/too hard) and relevance (6.8 agree/disagree).	Attitude	1 to 2A
Wiesner,^44^ 2018, Georgetown University School of Medicine, USA	Students answered precourse and postcourse knowledge-based tests with a maximum score achievable being 10. No outcomes were gathered on the skills based workshops teaching skills such as suturing and decontamination.	Students displayed significant improvement in their disaster medicine knowledge through completion of the course, with an improvement being demonstrated between mean precourse test score 5.3 (1.1) and mean postcourse test score 8.0 (1.0). The mean improvement in scores for all students on this course was 2.7 (p<0.0001, 95% CI 2.3 to 3.1).	Knowledge, skill	2B

### Level 1: trainee satisfaction

Trainee satisfaction was assessed in 13 (56.5%) studies and most commonly assessed using Likert type scales. Medical students were asked to rate the overall quality of the courses in addition to whether they would recommend courses to colleagues for disaster preparedness. Course satisfaction was generally very high and appeared to be enhanced by multimodal approaches to curriculum design including the incorporation of simulation and technology.^15^ However, one group discussion and interactive activity-based study did report mixed reviews with postcourse overall ratings of positive (33%), undecided (13%) and negative (54%). This is reflective of overburdening medical students with work, with one student stating, “This was way more work than it should have been. I would rather have an hour lecture on the flu than do all that group stuff. This was just frustrating to have at the end of the year when finals are right around the corner”.^16^

### Level 2A and 2B: trainee attitudes, knowledge and skill acquisition

Attitudes and perceptions of knowledge in medical students were assessed in 10 studies (43.5%). Attitudes were broadly assessed as level 2a following courses measuring either a simple change such as interest in disaster medicine, a measurement of a medical student’s willingness to volunteer in or preparedness to practice disaster medicine, or by mapping trainee responses to learning objectives. Level 2b was measured by a total of 20 studies (87.0%) which assessed medical student knowledge or skill acquisition, with 18 assessing knowledge and 11 assessing skills, either alone or in combination. In courses training students in mass casualty scenarios, discrete and measurable skills were easily assessed including tourniquet application and triage skills.^15^  ^17^

### Level 3: behavioural change

As behavioural change is a difficult area to measure in non-practicing medical students, only one study was deemed to adequately assess behavioural change. This study assessed confidence and perceived stress handling emergencies once the medical students had graduated 1.5 years following the course.^13^

### Level 4: clinical performance

No studies investigated the impact of disaster training on clinical performance (level 4a) or organisational delivery of care (level 4b).

### Risk of bias

**Figure 2:**
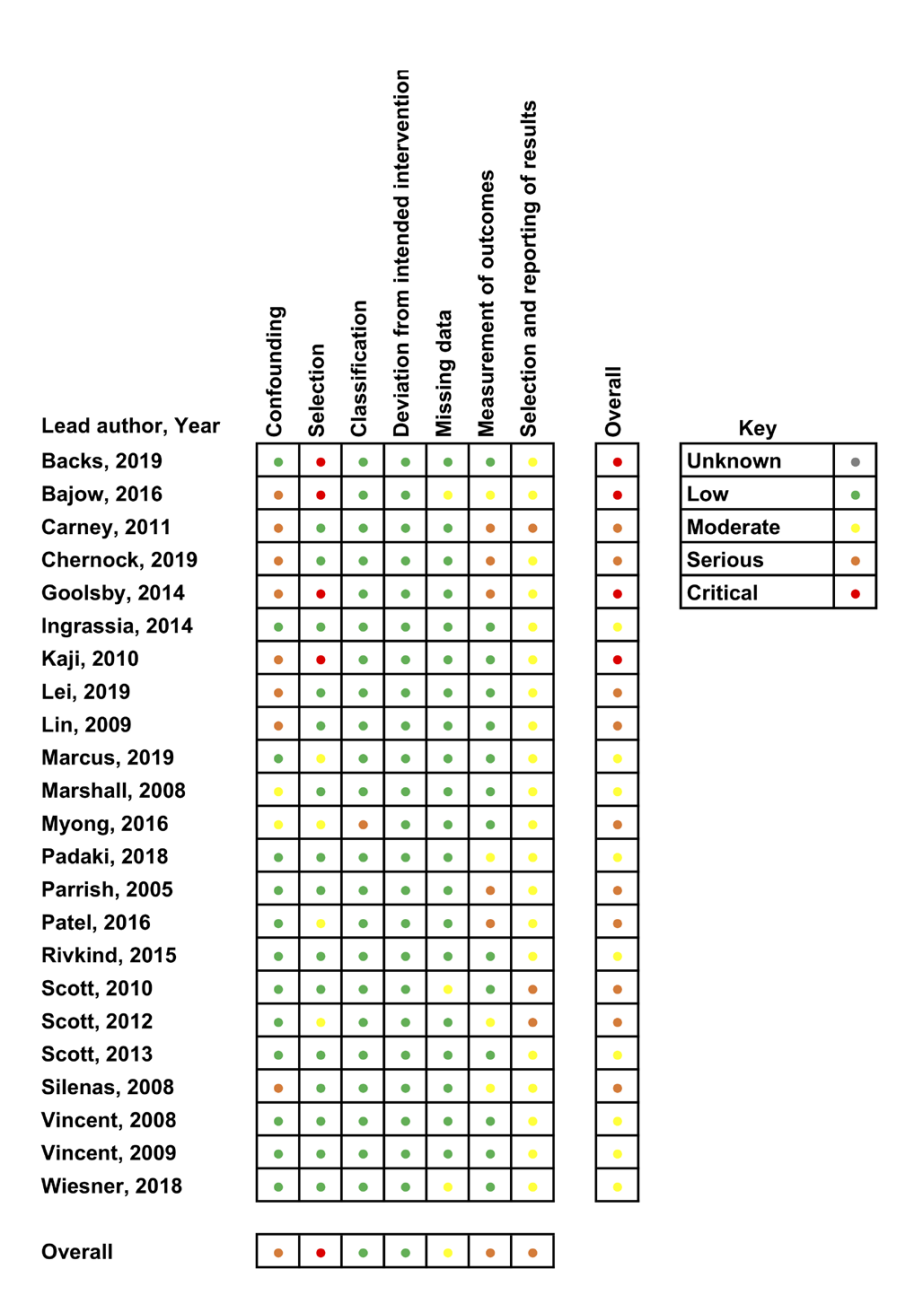
Individual risk of bias for non-randomised control trials determined by ROBINS-1.

**Figure 3:**
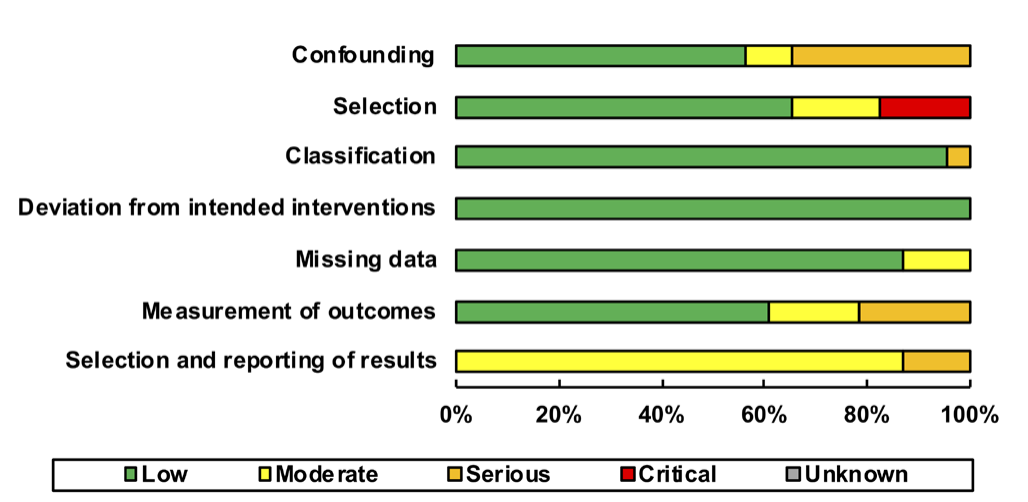
Overall risk of bias for non-randomised control trials determined by ROBINS-1.

All included studies were cohort studies and risk of bias was assessed using ROBINS-1 (figure 2).^12^ Risk of bias was low to critical (figure 3). Confounding bias was serious overall as many studies did not present pretest control data. There was critical overall bias in the selection of participants as courses were often not open to all students, for example, self-selected recruitment from student emergency medicine interest groups. Classification bias and bias due to deviation from intended interventions was low. A single study had a classification bias because they did not adequately describe their educational intervention—as all other studies had low bias, this domain was classified as having low overall bias.

Overall missing data bias was moderate as three studies had some form of missing data and were not able to adjust for this in their analysis. Measurement of outcomes had serious bias overall, as many questionnaire evaluations were subjective without any objective measures. Selection and report of results had serious bias overall. while some studies did note ethical approval there was no priori registration of the results and some studies had limited reporting of results.

## DISCUSSION

This systematic review identified approaches used to train medical students in disaster medicine in order to suggest training approaches for medical students in the current COVID-19 pandemic. We identified 23 studies published between 1996 and March 2020. Overall, medical student disaster training programmes improved student disaster and pandemic preparedness and resulted in improved attitude, knowledge and skills. There was an improvement in all studies that measured precourse and postcourse outcomes.

We found that all interventions ranging from simple classroom-based interactive discussion to complex multimodal simulative experiences resulted in improved knowledge, skill and attitudes towards participation in disaster medicine. The main outcomes of the courses reviewed were subjective; however, there was evidence to suggest that disaster medicine training does improve objective knowledge and can teach skills which can be used by medical students, relevant to a pandemic. The majority of courses were just 1 day in duration, indicating that short courses can still be impactful. The courses identified in this review required expert faculty or high-fidelity equipment and were implemented alongside an already busy medical school curriculum. These barriers prevented the majority of courses in this review from reaching longitudinal integration into medical school training. However, this may be overcome in the current COVID-19 pandemic by collaboration and coordination, particularly when many medical students have had their studies either postponed or converted to telemedicine/online teaching.

The main limitations of this review are related to study design, as the majority of studies were single centre and often focused on very specific aspects of disaster medicine. The overall reporting of both participant factors and outcome factors was generally poor, and the educational methodology was very heterogeneous—this was represented by critical risk of bias in selection of participants, and serious risk of bias in measurement of outcomes. This bias inevitably weakens the strength of the conclusions drawn, but given that all studies demonstrated a positive benefit, it can still be concluded that there will be benefit to students who undertake disaster preparedness courses.

Another limitation was the Kirkpatrick levels that were evaluated. Only one study evaluated change in behaviour (level 3) and no studies evaluated change in clinical performance (level 4a) or organisational patient benefit (level 4b). Furthermore, only three studies focused solely on pandemic influenza, airborne viral management or personal protective equipment (n=3, 13%), and only a single study assessed resuscitation in a disaster setting. This is of particular importance for the COVID-19 pandemic, where respiratory personal protective equipment is a necessity and there are specific resuscitation guidelines.^18^ Clinical impact and clinical utility must be taken into account when making suggestions for training during the COVID-19 pandemic.

Although medical students working during the COVID-19 pandemic will likely be deployed to non-infectious areas of work, there is no guarantee that medical students will not be exposed to the virus.^19^ Furthermore, a strain will be placed on healthcare services and contingency care may need to be provided in place of a traditional care service.^20^ Here, students may be essential in preserving the resilience of hospitals and community healthcare systems.^20^ There will ultimately be more pressure on medical students to work than previous cohorts and this review suggests that disaster medicine training as a part of medical school’s curriculum is not common practice. Therefore, medical students may require a very different set of competencies than those acquired during medical school. Unsurprisingly, some final year medical students do not feel ready to start as a newly qualified doctor, due to worries they are not well prepared for clinical placements, or feeling under prepared for COVID-19.^21^ Moreover, the Medical Schools Council have advised that medical students from any year should not take on roles that will impact on their studies.^8^

This review suggests that early mobilisation of medical students into the workforce could be accompanied by disaster medicine training. All courses reviewed in this study were positively evaluated by medical students, and if a similar programme was offered to current medical students, it would likely be well received improving willingness and preparedness to work in the healthcare service. This is of particular importance as medical students are already being asked to join the workforce as volunteers, or to graduate early in order to join healthcare systems as physicians. There is great concern that students who give assistance during a disaster without training are at an increased risk of both harm to themselves and psychological consequences.^22^ There is therefore a need to create novel courses to teach medical students pandemic skills in these unprecedented circumstances.

This review suggests that the most beneficial medical student disaster medicine courses should consist of mixed modalities of didactic sessions, case-studies, practical hands on training and simulation experiences.

### Suggested structure for COVID-19 training for medical students

These training methods could be used to train medical students in COVID-19 specific knowledge and skills and prepare them for clinical practice. Table 4 shows a proposed COVID-19 course and assessment based on the findings of this systematic review. The course structure includes the variety of elements found in other studies. Didactic lectures on COVID-19 could be delivered in a lecture hall with social distancing measures in place, or perhaps more appropriately as a distance learning component consisting of video, podcast and computer activities. Practical activities could include fitting of respiratory personal equipment as well as donning and doffing. The simulated element could consist of a patient with COVID-19 who requires cardiopulmonary resuscitation. In resource-limited scenarios, this could be undertaken using computer-based tutorials or video tutorials. As new doctors and medical students may have a substantial volume of information to learn in addition to this course, handouts and online refresher courses should be offered. The proposed assessment aims to cover all Kirkpatrick levels and criteria. It is also important to teach and train human factors awareness, particularly in relation to team dynamics, lowering authority gradients and empowering anyone to speak up if concerned.^23^ Maintaining both individual and team situational awareness is also important during any clinical duty, and even more so during a crisis setting.^23^ It may be useful to incorporate a credentialing process for medical students undergoing disaster training, thereby allowing students to demonstrate a background of competency and separating this cohort from unskilled volunteers when aiding a disaster medicine response.^24^ The successful implementation of these suggested disaster training techniques will require the encouragement of people-centred training, the development of peer-learning, coordination and funding of training systems, and regular disaster preparedness exercises of multimodality format.^25^

**Table 4:** Suggested COVID-19 course and assessment structure

Domain	Description
Course structure	COVID-19 training course for medical students consisting of: didactic lectures (with social distancing) or distance learning, eg, video, podcast and computer activities; case-based group discussion; practical activity, eg, respiratory personal protective equipment fitting; and high-fidelity simulation, eg, CPR for a patient with COVID-19
Medical student population	All medical students with priority given to medical students in their final year
Duration of intervention	1 day
Education setting	Lecture hall, classroom, indoor simulation (with social distancing or personal protective equipment), distance learning, online web system
Teaching methods	Lectures (with social distancing), practical skills, simulation, group discussion, computer activity, video, case study, handouts
Assessment	Knowledge—precourse and postcourse examination of didactic components assessing COVID-19 understanding Attitude—precourse and postcourse questionnaire on preparedness and willingness to perform duties Skill—summative assessment of practical activity and simulation Clinical—follow-up assessment over the following months assessing behavioural change and benefit to patients

CPR, cardiopulmonary resuscitation.

## Conclusion

The COVID-19 pandemic has caused unprecedented disruption to healthcare services in peacetime. Medical students may play a crucial role in the healthcare response. There is an imminent demand for educational interventions to train medical students to better assist in this response. The disaster medicine courses reviewed in this article improved knowledge, skills and attitudes through multimodal techniques and were well received by learners. Although no studies in this review demonstrated direct patient benefit, the courses increased student preparedness and similar courses should be implemented prior to medical students joining the healthcare workforce during the COVID-19 pandemic. Future courses should note the methodological and longitudinal flaws demonstrated in previous studies so that direct patient benefit can be demonstrated in the COVID-19 pandemic. Future work should be undertaken to ensure the successful integration of disaster training into global medical school curricula.

**Main messages**

- Medical students could play a crucial role in the SARS-CoV-2 healthcare response.
- Disaster medicine programmes using multimodal techniques improve knowledge, skills and attitudes which are imperative for medical practice in a pandemic.
- Training programmes incorporating previously successful techniques could ensure the successful integration of disaster training into global medical school curricula.

**Current research questions**

- Do disaster training programmes aimed at medical students demonstrate direct patient benefit?
- Can disaster training programmes improve the integration of medical students into the healthcare workforce during the SARS-CoV-2 pandemic?
- How can disaster training programmes be adapted to manage future pandemics?
